# Radioiodine Thyroid Remnant Ablation after Recombinant Human Thyrotropin or Thyroid Hormone Withdrawal in Patients with High-Risk Differentiated Thyroid Cancer

**DOI:** 10.1155/2012/481568

**Published:** 2012-12-06

**Authors:** Fabián Pitoia, Robert J. Marlowe, Erika Abelleira, Eduardo N. Faure, Fernanda Bueno, Diego Schwarzstein, Rubén Julio Lutfi, Hugo Niepomniszcze

**Affiliations:** ^1^Division of Endocrinology, Hospital de Clínicas, University of Buenos Aires, Córdoba 2351, 5th Floor, Buenos Aires 1424, Argentina; ^2^Division of Medical Editing, Spencer-Fontayne Corporation, 33 Bentley Avenue, Jersey City, NJ 07304-1901, USA; ^3^Division of Endocrinology, Hospital Churruca Visca, Uspallata 3400, Buenos Aires 1437, Argentina; ^4^Division of Endocrinology, Consultorios Integrados Rosario, Italia 424, Santa Fe, Rosario 2000, Argentina

## Abstract

To supplement limited relevant literature, we retrospectively compared ablation and disease outcomes in high-risk differentiated thyroid carcinoma (DTC) patients undergoing radioiodine thyroid remnant ablation aided by recombinant human thyrotropin (rhTSH) versus thyroid hormone withdrawal/withholding (THW). Our cohort was 45 consecutive antithyroglobulin antibody- (TgAb-) negative, T3-T4/N0-N1-Nx/M0 adults ablated with high activities at three referral centers. Ablation success comprised negative (<1 **μ**g/L) stimulated serum thyroglobulin (Tg) and TgAb, with absent or <0.1% scintigraphic thyroid bed uptake. “No evidence of disease” (NED) comprised negative unstimulated/stimulated Tg and no suspicious neck ultrasonography or pathological imaging or biopsy. “Persistent disease” was failure to achieve NED, “recurrence,” loss of NED status. rhTSH patients (*n* = 18) were oftener ≥45 years old and higher stage (*P* = 0.01), but otherwise not different than THW patients (*n* = 27) at baseline. rhTSH patients were significantly oftener successfully ablated compared to THW patients (83% versus 67%, *P* < 0.02). After respective 3.3 yr and 4.5 yr mean follow-ups (*P* = 0.02), NED was achieved oftener (72% versus 59%) and persistent disease was less frequent in rhTSH patients (22% versus 33%) (both comparisons *P* = 0.03). rhTSH stimulation is associated with at least as good outcomes as is THW in ablation of high-risk DTC patients.

## 1. Introduction

Postsurgical thyroid remnant ablation with radioiodine (131-iodine, ^131^I) in low-risk patients with differentiated thyroid carcinoma (DTC) has engendered considerable controversy [1]. However, current guidelines and consensus strongly favor the procedure in high-risk patients [2, 3]. 

 Thyroid-stimulating hormone (TSH) elevation is believed to be necessary to optimize ablative radioiodine uptake and organification [2]. The traditional method to obtain such elevation is endogenously, through thyroid hormone withdrawal or withholding (THW), with resultant hypothyroidism. An alternative to THW, available since 2001 in our country, Argentina, is exogenous TSH elevation via recombinant human TSH (rhTSH) administration [2, 4–8]. 

Numerous published comparisons [4, 9–16] have confirmed that rhTSH-aided ablation achieves high remnant eradication rates that are not statistically inferior to those attained with THW-assisted ablation. At the same time, relative to THW, rhTSH use avoids hypothyroid morbidity, improving patient quality-of-life [4, 14, 15, 17–19]. Compared to THW, rhTSH use also lessens extra-thyroidal radiation exposure [20, 21], improving safety [22]. Additionally, a number of published comparisons have documented statistically not different, modest DTC recurrence rates after rhTSH- or THW-aided ablation [9–11, 14, 16, 23]. rhTSH has a relatively high acquisition cost. However, the literature suggests that from the societal and patient/family perspectives, this cost may be balanced by the benefits of shorter hospital length-of-stay (where this variable is determined by whole-body dose rate), shorter absence from work, and improved on-the-job performance. These advantages are related to the preservation of euthyroidism and hence, of cognitive and physical function, when rhTSH is used [24–28]. One study also suggests that from an institutional perspective, the rhTSH acquisition cost may at least partly be offset by increased “patient throughput,” that is, more efficient use of radioiodine treatment rooms [28].

However, the preponderance of patients in publications regarding rhTSH-assisted versus THW-assisted ablation had low-intermediate postsurgical DTC recurrence risk; only two groups have published comparisons of the two modalities with respect to remnant eradication and disease persistence or recurrence focusing all or in part on high-risk DTC [9, 29–31]. 

The larger, more invasive primary tumors often characterizing high-risk disease might render complete cancer excision more difficult. Higher stage DTC also might be associated with increased risk of occult malignancy. Because of these challenges, it is important to compare outcomes in the postsurgical high-risk setting with rhTSH-aided versus with THW-aided ablation. We therefore undertook the present retrospective analysis.

## 2. Materials and Methods

### 2.1. Endpoints, Patients, and Ethics

 We examined rates of ablation success and of disease outcomes after medium-term follow-up according to the TSH preparation method for ablation in 45 consecutive adults ablated at any of three Argentine referral centers from March 2002 to June 2009. This cohort had initial T3-T4/N0-N1-Nx/M0 staging according to the American Joint Committee on Cancer/Union Internationale Contre le Cancer (AJCC/UICC) system, 6th edition [32], with undetectable antithyroglobulin antibodies (TgAb) by immunometric assay at the time of ablation. All T3 patients had gross invasion and the entire cohort had high recurrence risk according to the Latin American Thyroid Society (LATS) classification [3] and intermediate or high risk according to the American Thyroid Association (ATA) classification [2]. M0 status was confirmed by postablation whole-body scintigraphy (WBS). 

All patients were totally thyroidectomized in a specialized center. Thirty-six (80%) also underwent lymph node dissection. In 10 of the 36 (28% of the subgroup), central and lateral neck dissection was performed when intrasurgical anatomopathological frozen section analysis verified lymph node metastasis. In the remaining 26 of the 36 (72% of the subgroup), central neck (level VI) dissection was mostly indicated after T3 status confirmation, when suspicious lymph nodes were noted during surgery, or when both conditions pertained. 

Based on postsurgical histological analysis, 7 of these 26 patients eventually were found to have microscopic central lymph node metastasis. Therefore, of the 36 patients undergoing lymph node dissection, 17, or 47%, ultimately had confirmed nodal involvement.

Among the 45 patients in the overall cohort, ablation was aided by rhTSH (Thyrogen, thyrotropin alfa, Genzyme, Cambridge, MA, USA) in 18 (40%) and by THW in 27 (60%). The choice between rhTSH and THW was individualized according to physician and patient preferences and the patient's circumstances. Among the 18 rhTSH patients, indications for such preparation included poor general physical condition or advanced age (*n* = 6), patient preference (*n* = 6), generally due to desire to avoid hypothyroid morbidity or to decrease time missed from work or study, depression (*n* = 5), or cardiac disease (*n* = 1).

Tables 1(a) and 1(b) summarize key baseline patient characteristics by treatment group. rhTSH patients were on average a decade younger at DTC diagnosis than were their THW counterparts, a statistically significant difference. Nonetheless, the rhTSH group had a significantly greater proportion of patients ≥45 years old (Table 1(a)). Moreover, although there were no significant intergroup differences when T or N classifications were considered as individual categories for patients of all ages (Table 1(a)), patients aged ≥45 years tended to have more advanced T and N classifications, and thus, later AJCC/UICC stages, in the rhTSH group than in the THW group (Table 1(b)). The rhTSH patients were similar to their THW counterparts with respect to all other tested baseline variables.

Institutional review board approvals were obtained for the study. 

### 2.2. Ablation Protocol

 Our ablation protocol used fixed radioiodine activities based on the extent of initial disease, without adjustment according to the TSH preparation. Patients typically received 3.70 GBq (100 mCi) ^131^I for T3 disease with gross extension beyond the thyroid capsule and N0 status, 5.55 GBq (150 mCi) for T3/N1a-N1b disease, and 7.40 GBq (200 mCi) for T4 tumor. T4 patients (*n* = 12) received a second therapeutic ^131^I activity (mean ± standard deviation [SD] 3.37 ± 0.74 GBq [91 ± 20 mCi]), rhTSH-aided in all cases, a mean ± SD 9 ± 3 months after ablation. A low-iodine diet was prescribed from one week before radioiodine administration through two days afterwards. Pretherapeutic urinary iodine testing was not routine; however, patients were queried about exposure to possible sources of iodine excess, which was not reported in any case, but would have been grounds to delay ^131^I administration.

 To reduce the risk of actinic thyroiditis from administering large amounts of radioiodine to bulky residues, we assessed preablation thyroid remnant size in some patients (*n* = 6) operated on by surgeons with whose thyroid procedure expertise we were unfamiliar. In those patients, a 3.7 MBq (100 *μ*Ci) ^131^I tracer activity was given the day preceding, and cervical percentage uptake was ascertained just before ablative activity administration [33]. Uptake above our 3%–5% norm would have warranted consideration of a reduced ablative activity or referral for reoperation, but was not seen.

Posttherapy WBS was performed 5–7 days after ablation and any second radioiodine therapy.

### 2.3. TSH Preparation

 rhTSH was given as two consecutive daily 0.9 mg intramuscular injections, with the tracer activity (when applied) administered at the time of the second injection and the ablative activity or second radioiodine therapy (T4 patients only) administered ~24 hr after the second injection. rhTSH was given while patients were euthyroid with suppressed TSH and receiving levothyroxine, except that this hormone was briefly withdrawn, from 2 days before through 2 days after radioiodine therapy administration, to reduce the risk of iodine interference [34].

THW comprised at least 3 weeks without thyroid hormone, starting from thyroidectomy. Radioiodine was administered following that interval, in all cases with TSH levels above 50 mIU/L. 

### 2.4. Thyroglobulin (Tg)/TgAb Measurement

Samples for Tg and TgAb measurement were taken on day 5 after the first rhTSH injection in the rhTSH group and on the day of ablative radioiodine administration in the THW group. Tg and TgAb levels were assessed in one of three reference laboratories (depending on the center), using either of two commercial immunometric assays; the same laboratory and assay were used throughout a patient's follow-up. Tg assays comprised the Elecsys Tg Electrochemiluminescence Immunoassay (Roche Diagnostics GmbH, Mannheim, Germany), which has a 0.5 *μ*g/L detection limit, or the Immulite 2000 Tg Chemiluminiscence Assay (Siemens Corp., Los Angeles, CA, USA), with a 0.2 *μ*g/L analytical sensitivity. TgAb assays comprised the Elecsys Anti-Tg Electrochemiluminescence Immunoassay (RSR Ltd., Pentwyn, Cardiff, UK), or the Immulite 2000 Anti-TG Ab chemiluminescent immunometric assay method (Siemens). For both TgAb assays, values >20 IU/mL were considered to be positive, and to render Tg measurements uninterpretable.

### 2.5. Follow-Up Including Ablation Success Assessment

**Figure 1:**
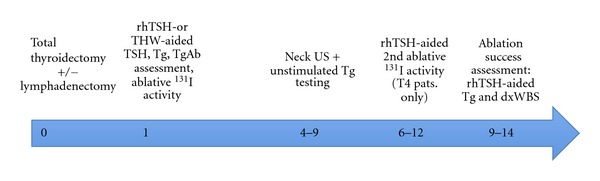
Initial treatment and follow-up regimen in patients with thyroid cancer included in the study. Thyroid surgery represents time zero; other numbers refer to months after surgery. Dx, diagnostic; rhTSH, recombinant human thyrotropin; Tg, thyroglobulin; TgAb, antithyroglobulin antibodies; TSH, thyroid-stimulating hormone; US, ultrasonography.

Figure 1 represents our protocol for initial treatment and follow-up. After ablation, all patients started (THW group) or restarted (rhTSH group) suppressive thyroid hormone therapy (target TSH: <0.01 mIU/L). 

Ablation status was assessed using rhTSH-stimulated Tg testing and rhTSH-stimulated diagnostic WBS (dxWBS; 150 MBq [4 mCi] activity) performed 6–12 (mean ± SD 9 ± 4) months after ablation. Ablation success was defined as negative stimulated Tg (<1 *μ*g/L) in the absence of TgAb, plus absent or <0.1% thyroid bed uptake on dxWBS.

Neck ultrasonography (US) using an 11 MHz linear array transducer was performed every 6 months after ablation. Patients with measurable stimulated or unstimulated Tg, suspicious neck US findings, or both during follow-up underwent morphological or functional imaging or both, including computed tomography (CT) (*n* = 7 [39%] in the rhTSH group; *n* = 13 [48%] in the THW group) or 18-fluorodeoxyglucose positron emission tomography (FDG-PET) (*n* = 6 [33%] in the rhTSH group; *n* = 4 [15%] in the THW group). All ultrasonographically suspicious nodules ≥1 cm in diameter underwent fine needle aspiration with measurement of Tg in the aspirate.

### 2.6. Disease Status Definitions

We defined disease status according to the latest LATS [3] and ATA guidelines [2]. Patients had “no evidence of disease” (NED) when unstimulated and stimulated Tg were negative (<1 *μ*g/L), TgAb were negative (<20 IU/mL), neck US was free of suspicious signs, and there were no pathological findings on any other imaging (WBS, radiography, CT, FDG-PET, or any other modality) or in any biopsy specimen. However, patients with a single stimulated Tg measurement ≥1 to ≤2 *μ*g/L without additional signs of DTC were considered to have indeterminate status until a subsequent stimulated Tg measurement became negative or exceeded 2 *μ*g/L; the latter increase was considered a sign of disease. Patients who never attained NED status were classified as having “persistent disease,” while those who lost NED status were defined as having “recurrent disease.” Disease sites were classified as local (thyroid bed), lymph node (metastasis confirmed by fine-needle aspiration biopsy with positive cytology), distant (metastasis confirmed by imaging), or unknown (“biochemical only”) (stimulated Tg >2 *μ*g/L without structural evidence of disease).

### 2.7. Statistics

Data are expressed as mean ± SD unless otherwise noted. Categorical comparisons were made using chi-square testing with the Fisher's exact test when appropriate. Analysis was performed using SPSS software (version 15.0.0: SPSS, Inc., Chicago, IL, USA). *P* values  ≤0.05 were considered to be statistically significant. 

## 3. Results

Table 2 provides key data regarding ablation and ablation success for both treatment groups. On average, the treatment groups did not differ regarding the ablative activity, the proportions of patients in different activity categories or receiving a second therapy, the cumulative activity, or the interval between ablation and ablation success assessment. However, successful remnant ablation was observed in a significantly greater proportion of the rhTSH group than of the THW group (83% versus 67%, *P* = 0.02). 

Table 3 highlights the disease status at the end of follow-up according to the treatment group. The mean duration of follow-up was approximately 3 1/3 years in the rhTSH group and a year longer than that in the THW group, a statistically significant difference (*P* = 0.02). The rhTSH patients achieved NED status significantly more often and showed persistent disease significantly less often than did their THW counterparts. The groups did not differ with respect to recurrence rates. No patient developed TgAb or received more than the planned one (T3 patients) or two (T4 patients) ablative radioiodine therapies during follow-up.

 Sites of persistence and recurrence appeared to have a roughly similar distribution in the treatment groups; all recurrences affected lymph nodes, and there was no structural evidence of distant recurrence. The times to recurrence detection were 22 months in the rhTSH patient and 19 and 20 months, respectively, in the THW patients who lost NED status. Patients with neck lymph node metastases or recurrence in the neck lymph nodes were reoperated. The patients were reevaluated at 6 ± 4 months after reoperation with rhTSH-stimulated Tg testing and all had biochemical persistence without structural evidence of disease at the latest follow-up.

## 4. Discussion

This retrospective analysis in DTC patients with an high recurrence risk but no known distant metastases had three main findings regarding rhTSH versus THW stimulation of radioiodine thyroid remnant ablation. rhTSH use was associated with firstly, a significantly higher ablation success rate, and secondly, significantly more frequent favorable medium-term disease outcomes, that is, more frequent NED status and less frequent persistent disease. Thirdly, despite our cohort's high-risk status, both TSH preparation methods appeared to be associated with modest medium-term recurrence rates that did not differ statistically. It is worth noting the context of these observations: more than double the proportion of the rhTSH group than of the THW group was ≥45 years old (78% versus 37%, Table 1(a)), and the rhTSH patients ≥45 years old tended to have more advanced T and N stages than did their THW counterparts (Table 1(b)), even though the groups did not differ significantly when T or N stages were considered as individual categories for patients of all ages (Table 1(a)).

Our findings confirm those of the Santa Casa do Belo Horizonte [9] and Memorial Sloan Kettering Cancer Center (MSKCC) groups [29–31] of numerically similar or statistically not different or superior ablation success and disease outcome rates associated with rhTSH versus THW preparation of ablation in initial high-risk DTC patients. Regarding ablation success, the Belo Horizonte investigators noted respective 80% versus 79% rates (68% and 67% in patients with Tg > 1 *μ*g/L at ablation) in the rhTSH (*n* = 77) and THW subgroups (*n* = 198) of a slightly lower risk cohort than ours (T3/N0-N1 but no T4 patients) [9]. The MSKCC investigators reported respective 16% versus 9% rates of “excellent” response to initial therapy and respective 13% versus 7% rates of “acceptable” response in their rhTSH (*n* = 69) and THW subgroups (*n* = 92) of patients with initial ATA high-risk classification; these rates did not significantly differ [30]. Our ablation success rates were 83% for the rhTSH group (*n* = 18) versus 67% for the THW group (*P* = 0.02) (Table 2). It should be noted that the MSKCC “response to initial therapy” variable encompassed a longer postablation follow-up (2 years) than did our or the Brazilian investigators' “ablation success” variables (6–12 and 9–12 months, resp.). Additionally, both the Belo Horizonte and MSKCC groups included ultrasonographic findings in their assessment of response to ablation or “initial treatment,” while we did not. 

With respect to disease outcome, the Belo Horizonte investigators observed numerically similar, low 9–12-month disease persistence rates in rhTSH patients (*n* = 70) and THW patients (*n* = 169) whose M0 status was confirmed by the postablation WBS: 7.1% versus 7.7%. After a presumably much longer follow-up (median not reported for the initial ATA high-risk patients, but 9 years for their overall cohort [*N* = 586]), the MSKCC group noted statistically not different disease outcomes for rhTSH and THW patients with initial ATA high-risk status: 17.1% NED, 82.6% disease persistence, and 0% recurrence rates for the rhTSH patients versus 15.2%, 83.7%, and 1.1%, respectively, in their THW counterparts. In our study sample, after a mean follow-up of ~3.3 years in the rhTSH patients and ~4.5 years in the THW patients, the NED rate was significantly higher (72% versus 59%, *P* = 0.03) and the disease persistence rate was significantly lower (22% versus 33%, *P* = 0.03) in the rhTSH group (Table 3). The differences in ablation success and disease outcomes among the Belo Horizonte, MSKCC, and our groups may be attributable to one or more of (1) different follow-up durations; (2) noninclusion of US findings among our ablation success criteria; (3) lack of T4 patients in the Belo Horizonte group; (4) inclusion of only N1 patients with especially extensive neck nodal involvement in the MSKCC cohort; (5) somewhat higher (by ~700 MBq, ~19 mCi) mean cumulative ^131^I activities in our patients than in their Belo Horizonte counterparts—and perhaps also the MSKCC initial ATA high-risk patients (mean cumulative activity not reported for the latter subgroup, but ~500 MBq [~14 mCi] lower in the overall MSKCC study sample than in our cohort) [9, 30].

 Limitations of our study should be noted. Among these were its retrospective nature (shared with the Belo Horizonte and MSKCC studies), relatively small patient cohort, and discrepant mean follow-up durations for the treatment groups. Regarding the first of these limitations, our rhTSH and THW patients nonetheless had quite similar tested baseline and treatment characteristics; key characteristics that differed significantly (proportion of patients ≥45 years old, AJCC/UICC stage) presumably would have favored the THW group. Regarding the follow-up length, which was on average significantly shorter in rhTSH patients (Table 3), one would expect this difference to be relevant mainly to the observed DTC recurrence rate, which did not differ between the treatment groups, rather than to the NED or disease persistence rates, which differed in favor of rhTSH. 

## 5. Conclusion

In a cohort of adult referral center patients with LATS and ATA initial high-risk DTC without known distant metastases, rhTSH-stimulated radioiodine thyroid remnant ablation was associated with significantly greater ablation success rates than was THW-aided ablation. Additionally, after medium-term follow-up, rhTSH stimulation was associated with significantly more frequent NED status, significantly less frequent disease persistence, and statistically not different, low DTC recurrence rates. These results suggest that patients and clinicians do not have to consider initial disease classification in their choice of TSH preparation for postsurgical ablation in DTC without distant metastasis; these observations should be prospectively confirmed. 

## Figures and Tables

**Table:** (a)

Variable	rhTSH group (*n* = 18)	THW group (*n* = 27)	*P *
Age at DTC diagnosis			
Mean ± SD years	41 ± 16	51 ± 14	0.03
≥45 years old, % (*n*)	78% (14)	37% (10)	0.01

Female, % (*n*)	83% (15)	78% (21)	0.30

Histological classification, % (*n*)			
Papillary, classical variant	78% (14)	82% (22)	
Papillary, follicular variant	6% (1)	11% (3)	0.08
Follicular	16% (3)	7% (2)	

T classification^a^, % (*n*)			
T3	72% (13)	74% (20)	
T4a	22% (4)	19% (5)	0.53
T4b	6% (1)	7% (2)	

N classification^a^, % (*n*)			
N0/Nx	67% (12)	66% (16)	
N1a	27% (5)	22% (6)	0.08
N1b	6% (1)	18% (5)	

Patients with vascular invasion, % (*n*)	22% (4)	26% (7)	0.33

AJCC/UICC stage^ab^, % (*n*)			
I	22% (4)	63% (17)	0.01
II	0% (0)	0% (0)	
III	50% (9)	33% (9)	
IVa	11% (2)	4% (1)	
IVb	17% (3)	0% (0)	
IVc	0% (0)	0% (0)	

Lymph node dissection, *n* (%)	78% (14)	81% (22)	0.49

AJCC: American Joint Committee on Cancer; DTC: differentiated thyroid carcinoma; rhTSH: recombinant human thyroid-stimulating hormone; SD: standard deviation; UICC: Union Internationale Contre le Cancer; THW: thyroid hormone withdrawal or withholding.

^a^According to AJCC/UICC classification, 6th edition of 2002 [32]; all patients had M0 staging confirmed by the postablation whole-body scan.

^b^See Table 1(b) for explanation of the basis of staging in this cohort.

**Table:** (b)

TN status^ a^, *n *	AJCC/UICC stage^a^	rhTSH group (*n* = 18)	THW group (*n* = 27)
Age < 45 years			

T3N0	I	2	3
T3Nx	I	0	2
T3N1a	I	2	2
T3N1b	I	0	4
T4aN0	I	0	4
T4bN1a	I	0	1
T4bN1b	I	0	1

Age ≥ 45 years			

T3N0	III	7	4
T3Nx	III	0	2
T3N1a	III	2	3
T4aN0	IVa	0	1
T4bN0	IVb	3	0
T4bN1a	IVa	1	0
T4bN1b	IVa	1	0

AJCC: American Joint Committee on Cancer; DTC: differentiated thyroid carcinoma; rhTSH: recombinant human thyroid-stimulating hormone; UICC: Union Internationale Contre le Cancer; THW: thyroid hormone withdrawal or withholding.

^a^According to AJCC/UICC classification, 6th edition of 2002 [32]; all patients had M0 staging confirmed by the postablation whole-body scan.

**Table 2:** Ablation characteristics by patient group.

Variable	rhTSH group (*n* = 18)	THW group (*n* = 27)	*P *
Cumulative radioiodine activity before ablation success evaluation, GBq (mCi), mean ± SD	5.62 ± 1.30 (152 ± 35)	5.85 ± 1.22 (158 ± 33)	0.34

Ablation activity, GBq (mCi), mean ± SD	4.70 ± 1.41 (127 ± 38)	4.85 ± 1.11 (131 ± 29)	0.32

Ablation activity category, *n *			
3.70 GBq (100 mCi)	9	11	
5.55 GBq (150 mCi)	4	9	0.08
7.40 GBq (200 mCi)	5	7	

Patients receiving 1 additional radioiodine therapy before ablation success evaluation, % (*n*)	28% (5)	26% (7)	0.43

Timing of ablation success evaluation, months after ablation, mean ± SD	10 ± 3	9 ± 5	0.18

Ablation status, % (*n*)			
Success	83% (15)	67% (18)	0.02
Failure	17% (3)	33% (9)	

dxWBS: diagnostic whole-body scintigraphy; rhTSH: recombinant human thyroid-stimulating hormone; rxWBS: posttherapy whole-body scintigraphy; SD: standard deviation; Tg: serum thyroglobulin; THW: thyroid hormone withdrawal or withholding; TSH: thyrotropin.

**Table 3:** Disease status at the end of followup.

Variable	rhTSH group (*n* = 18)	THW group (*n* = 27)	*P *
Postablation followup, months, mean ± SD	40 ± 16	54 ± 40	0.02

Disease status^a^ at the end of followup, % (*n*)			
NED	72% (13)	59% (16)	0.03
Persistent disease	22% (4)	33% (9)	0.03
Recurrent disease	6% (1)	7% (2)	NS

Sites of persistent disease, *n *			
Local	0	2	Not tested
Lymph nodes	1	3	
Unknown (biochemical only)^b^	3	4	

Sites of recurrence, *n *			
Local	0	0	Not tested
Lymph nodes	1	2	
Unknown (biochemical only)^b^	0	0	

NED: no evidence of disease; rhTSH: recombinant human thyroid-stimulating hormone; SD: standard deviation; THW: thyroid hormone withdrawal or withholding.

^a^Please refer to “Materials and Methods” section for a description of potential disease states.

^b^Refers to elevated stimulated Tg (>2 *μ*g/L) without structural evidence of disease.
